# Synthesis and Characterization of Acrylic Resin/Kaolin Composites for Dielectric Applications

**DOI:** 10.3390/polym16233376

**Published:** 2024-11-29

**Authors:** Dorel Buncianu, Eduard-Marius Lungulescu, Alina Caramitu, Virgil Marinescu, Liviu Marsavina, Erwin-Christian Lovasz

**Affiliations:** 1 Faculty of Mechanics, University Politehnica of Timisoara, 2 Piata Victoriei, 300006 Timisoara, Romania; dorel.buncianu@upt.ro (D.B.); liviu.marsavina@upt.ro (L.M.); ervin.lovasz@upt.ro (E.-C.L.); 2 National Institute for Research and Development in Electrical Engineering ICPE-CA, 313 Splaiul Unirii, 030138 Bucharest, Romania; alina.caramitu@icpe-ca.ro (A.C.); virgil.marinescu@icpe-ca.ro (V.M.)

**Keywords:** acrylic resin composites, kaolin filler, thermal stability, dielectric strength

## Abstract

This study investigates the synthesis and characterization of acrylic resin/kaolin composites for dielectric applications. Acrylic resin, while widely used for its mechanical strength and ease of processing, exhibits limited dielectric properties, which restrict its use in high-performance electrical insulation. To address this, varying concentrations (0–70%) of raw kaolin, containing 71% kaolinite, were incorporated into an acrylic resin matrix to enhance its dielectric strength and thermal stability. Characterization techniques such as Fourier-transform infrared spectroscopy (FTIR), scanning electron microscopy (SEM), differential scanning calorimetry (DSC), and dielectric spectroscopy were used to analyze the molecular structure, morphology, thermal behavior, and dielectric properties of the resulting composites. The study found that with up to 30% kaolin, the composites demonstrated good dielectric performance and thermal resistance, with good particle dispersion and minimal agglomeration. However, beyond 30% filler content, the dielectric and mechanical properties began to decline drastically. The results suggest that these composites could be potentially used for moderate dielectric applications such as insulators and capacitors.

## 1. Introduction

Polymeric composites have become essential materials in modern engineering and technology. They offer a unique combination of lightweight and versatility properties, mechanical flexibility, recyclability, high electrical breakdown strength, and the ability to be customized for specific applications (e.g., automotive, construction, packaging, aerospace, electronics, etc.) [1,2,3]. Among polymer composites, thermosetting resins, such as epoxy, polyester, and phenolic resins, are the most commonly used for applications requiring excellent electrical insulating properties due to their unique characteristics: (i) epoxy resins provide high strength and chemical resistance, making them ideal for electronics and electrical components [4,5,6]; (ii) polyester resins, known for their cost-effectiveness and ease of processing, are widely used in the construction of electrical panels and insulating materials [7]; (iii) phenolic resins offer exceptional thermal stability and flame resistance, making them suitable for high-temperature applications and electrical insulators [8].

Acrylic resin, which is a versatile polymer, has been used in various industrial applications due to its excellent mechanical strength, flexibility, elasticity, and ease of processing [9,10]. Although acrylic resins have many benefits, some high-performance applications (e.g., high-frequency electronic components, and capacitors) may not be able to use them due to their comparatively low dielectric constant [11]. To overcome these limitations, researchers have extensively explored incorporating various fillers into the polymer matrix. These fillers enhance the dielectric strength and thermal stability of the resin, making it more suitable for demanding electrical applications [10,12].

Factors including the type, size, shape, and distribution of fillers, together with their interfacial interactions with the polymer matrix, represent an extremely complex field in studies of polymer matrix/filler interactions, and influence the properties of the resulting composites collectively. Filler materials, properly dispersed, may enhance the tensile strength and modulus of composites by significantly providing more surface area for interaction because of their smaller sizes, thus improving mechanical properties [13]. It is also important to have a uniform distribution of fillers to ensure effective transfer of load between the matrix and the filler, thus reducing stress concentrations that may cause failure in load [14]. Studies have proven that size optimization of filler particles and ensuring homogeneity within the matrix maximize the strength and toughness of composites [13,15]. Also, filler shape has also been reported to play a role in stress transfer and polymer chain mobility [16]. Optimizing these factors might provide paths to high-performance materials for a wide variety of applications.

Kaolin, which is a naturally occurring aluminosilicate clay, is widely utilized across various industries due to its unique properties. Renowned for its whiteness, purity, and fine particle size, kaolin serves as an important component in ceramics, paper, paint, rubber, and pharmaceuticals, among others [17]. Its exceptional characteristics make it a valuable filler, coating, and absorbent material [18,19]. The use of kaolin as a filler in a polymer matrix leads to an improvement in the resulting composite’s mechanical, thermal, barrier, and dielectric properties. Both the use of small particle size and their favorable surface chemistry (due to the presence of functional groups, such as hydroxyl groups, which can form hydrogen bonds or other intermolecular interactions with polymer chains) lead to improved adhesion and interfacial bonding, facilitating a uniform dispersion within the polymer matrix. These factors are essential for achieving the desired dielectric strength of the composite [19,20,21].

This study investigates obtaining and characterizing KM-U acrylic resin/kaolin composites with a focus on their dielectric, mechanical, and thermal behavior properties, aiming to elucidate the impact of kaolin content on these properties. By systematically varying filler concentration and analyzing the resulting material properties, we seek to identify the optimal composite formulation for use in advanced dielectric applications.

The significance of this research lies in its potential to contribute to the development of low-cost dielectric materials with tailored properties for specific applications. These materials could open the way for new dielectric insulators, offering enhanced performance, reliability, and cost-efficiency.

## 2. Materials and Methods

### 2.1. Materials

The composite matrix was a green, unsaturated acrylic resin powder (KM-U, PRESI, Eybens, France). The raw kaolin BIP powder material (Imerys, Vaulx-Milieu, France), comprising 71% kaolinite according to the producer, was used as the second dispersed phase in the acrylic resin. Its chemical composition has been detailed in a prior study [6]. The initial powder, with a coarse particle size of around 150 μm, was refined to improve its properties. To achieve this, the kaolin powder was wet-ground for 5 h at 60 rpm in water to minimize heat generation and prevent agglomeration. The ground powder was then dried in an oven at 100 °C for 24 h to remove residual moisture. Finally, the dried kaolin was sieved through a 40 μm sieve before being incorporated into KM-U resin.

### 2.2. Obtaining Acrylic Resin/Kaolin Composites

**Figure 1:**
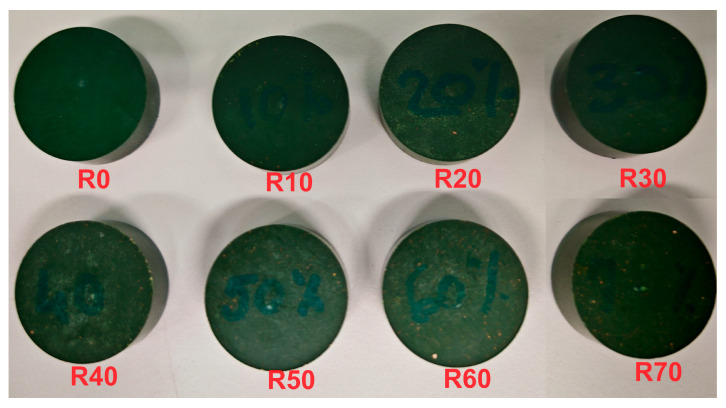
KM-U resin/kaolin composite samples (sample codes: R0–R70, where the numbers represent kaolin amount in %).

The process for obtaining resin/kaolin composite materials involves several phases. Initially, the resin particles were ground using a ceramic ball mill at 500 rpm for 30 min to ensure a homogeneous distribution in the resin/kaolin composites. The ground material was then sieved through a 40 µm sieve to standardize particle sizes. Next, the sieved resin powder was mixed with kaolin in varying concentrations (0% to 70% by mass), with 10× *g* of resin powder being gradually combined with the respective amounts of kaolin. This mixture was homogenized in a plastic container using a wooden spatula, maintaining a controlled environment to prevent contamination. Finally, the homogenized mixture was formed into disks with a diameter of 30 mm (Figure 1) by subjecting it to a pressure of 9000 N (Newton) for 170 s at 180 °C, followed by a recovery/cooling period of 180 s.

### 2.3. Characterization of Acrylic Resin/Kaolin Composites

#### 2.3.1. Fourier-Transform Infrared Spectroscopy (FTIR)

FTIR spectra of the resin/kaolin composite samples were recorded using a Jasco 4200 spectrometer (Jasco Inc., Tokyo, Japan) coupled with a JASCO PRO 470-H ATR (Attenuated Total Reflectance) accessory. The samples were measured directly by placing them on the ATR device’s crystal and pressing with a controlled force. The spectra were recorded over a spectral range of 4000–500 cm^−1^ with a resolution of 4 cm^−1^ and 50 scans per spectrum.

#### 2.3.2. Thermal Analysis

The thermal properties of the samples were analyzed by using differential scanning calorimetry (DSC) and Thermogravimetric/Differential Thermal Analysis (TG/DTA). DSC analysis was performed on a Setaram DSC 131 EVO (Paris, France) to evaluate its response to temperature changes. Approximately 4 mg of sample was placed in a 30 μL aluminum crucible and heated in an air atmosphere (50 mL/min) from 30 °C to 500 °C at a heating rate of 10 °C/min.

TG/DTG analysis was conducted with a NETZSCH STA 449 F3 Jupiter simultaneous thermal analyzer (NETZSCH, Selb, Germany) over a temperature range of 35 to 900 °C in alumina (Al_2_O_3_) crucibles. The heating rate was set to 10 °C per minute, and the analysis was performed under a nitrogen atmosphere to ensure an inert environment.

#### 2.3.3. Scanning Electron Microscopy (SEM)

The morphology of the samples was investigated using a hot field emission gun scanning electron microscope (FE-SEM) model Auriga, manufactured by Carl Zeiss (Oberkochen, Germany). The instrument was operated at an accelerating voltage of 10 kV and a working distance of 5 mm. Scanning electron microscopy (SEM) images were acquired on uncoated samples using a local Charge Compensation (CC) system with nitrogen gas and a secondary electron detector (SE) in an Everhart–Thornley detector chamber. The SEM images of the composite disc-shaped samples were captured at their fracture surfaces.

#### 2.3.4. Electrical Properties

The dielectric properties of composites, including the tangent of dielectric loss angle (tan δ; dissipation factor) and dielectric constant (ε′), were measured using dielectric spectroscopy. A Solartron 1260A dielectric spectrometer (Solartron Analytical, Farnborough, UK) was employed at an AC voltage of 3 V across a frequency range of 1–1000 kHz. A measuring electrode with a diameter of 30 mm was used for the analysis. The analyzed samples had a diameter of 30 mm, while the thickness varied between 10 and 13 mm, depending on the kaolin percentage. The dielectric measurements were performed at room temperature.

**Figure 2:**
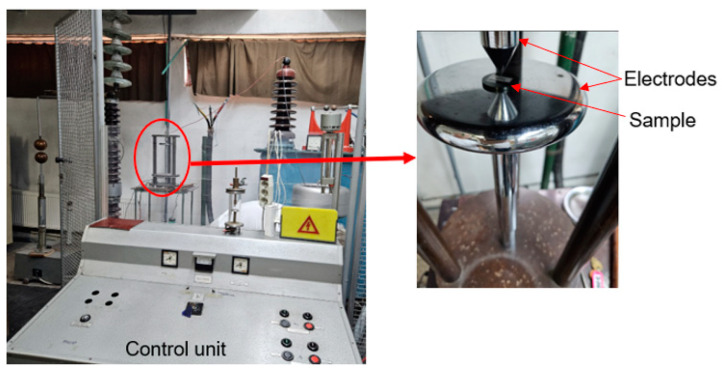
Dielectric breakdown strength testing setup.

The dielectric breakdown strength of the samples was determined using a high-voltage testing apparatus in accordance with ASTM D149-20 [22]. The tests were conducted under laboratory conditions at a frequency of 60 Hz, with a voltage rate of rise of 0.5 kV/s. A custom-built testing setup, as depicted in Figure 2, was employed for these experiments.

In the initial phase of dielectric testing, plate-type electrodes were used for both samples. After the first trials, the occurrence of contouring was observed at very low voltages. In the first stage, the upper plate electrode was replaced with a sharpened electrode to concentrate the electric field; subsequently, both electrodes (upper and lower) were substituted with sharpened ones. Measurements were conducted using sharpened electrodes for all samples, adhering to the same protocol. All samples were mechanically processed to standardized dimensions in accordance with standard operating norms (diameter: 30 mm, height: 2 mm).

#### 2.3.5. Mechanical Properties

A diametral compression test, known as a Brazilian disc test [23], was adopted to determine tensile strength. Disk specimens were loaded with a uniform pressure, which was radially applied over a short strip on the circumference at each end of a diameter. The frictional stresses between loading platens and specimen were neglected. The tests were performed using a universal testing machine, Zwick Z005 (Zwick/Roell, Ulm, Germany), having maximum load of 5 kN. Tests were performed at room temperature (i.e., 22 °C) with a loading speed of 5 mm/min. Three tests were performed for each kaolin content.

The tensile strength was determined with the following relation [24]:

(1) $$\sigma_{t}=\frac{2P}{\pi DB}$$

where P represents the maximum compression load [N]; D is the disk diameter [mm]; and B is the disk thickness [mm].

## 3. Results and Discussion

### 3.1. SEM Microscopy

Compared to liquid resins, resin powder forms exhibit lower viscosity, allowing for easier handling and dispersion within a matrix, leading to improved homogeneity and reduced void formation, and enhanced mechanical properties of the final composite [6]. Additionally, powder resins often have lower shrinkage during curing, minimizing internal stresses and deformations. Their dry form also eliminates the need for volatile organic compounds (VOCs) associated with liquid resins, making them a more environmentally friendly option [25,26].

**Figure 3:**
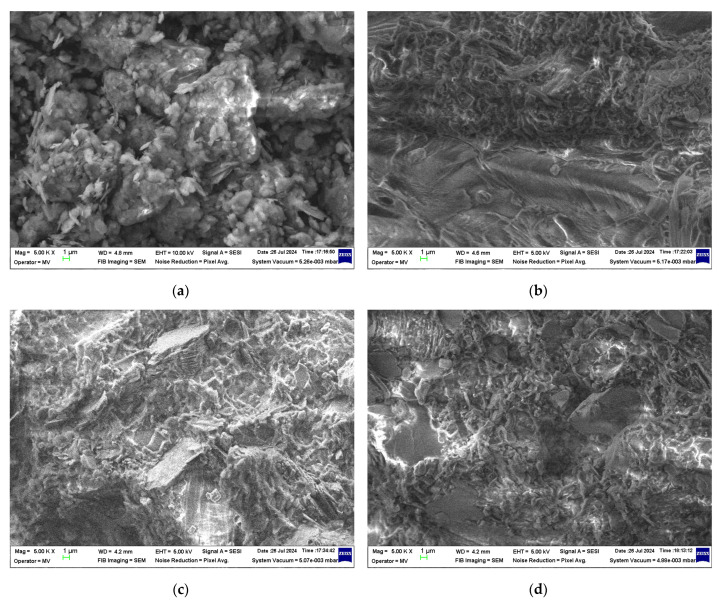
SEM images recorded on (**a**) kaolin; (**b**) R0; (**c**) R30; (**d**) R70.

Figure 3a–d present SEM images illustrating the morphology of kaolin powder and KM-U/kaolin composites at varying concentrations (R0, R30, and R70). The kaolin particles are characterized by their acicular shape and a tendency to form agglomerates. In contrast, the SEM images of KM-U/kaolin composites reveal a well-distributed dispersion of kaolin particles within the acrylic resin matrix, with no noticeable agglomeration observed, even at the highest kaolin concentration (R70). This suggests that the incorporation of kaolin into acrylic resin effectively mitigates particle clustering, achieving a uniform composite structure.

### 3.2. FTIR Spectroscopy

**Figure 4:**
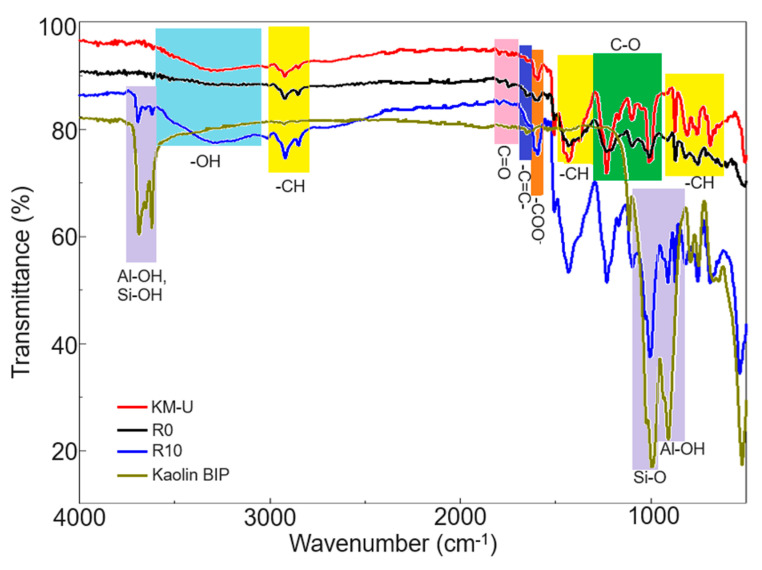
FTIR spectra of KM-U resin/kaolin composites.

The characteristic bond vibrations of kaolin (Figure 4) were identified at specific wavenumbers, each corresponding to a distinct type of molecular motion within the structure. These include 456 cm^−1^ for Si-O-Si bending, 571 cm^−1^ for Si-O-Al stretching, 791 cm^−1^ for Al-OH deformation, 908 cm^−1^ for Al-OH bending, 950–1200 cm^−1^ for Si-O stretching, 1600–1660 cm^−1^ for the bending of intercalated H-O-H, and 3686, 3650, and 3620 cm^−1^ for various -OH stretching vibration modes specific to kaolinite (Al-OH and Si-OH stretching [27,28]). The presence of a very low-intensity band in the vibrational range of the –CH groups (2800–2900 cm⁻^1^) in the spectral analysis suggests minimal contamination with organic compounds, indicating that the kaolin powder used in the synthesis of KM-U/kaolin composites is of high purity.

The FTIR spectrum of KM-U powder resin reveals several key functional group vibrations. A broad O-H stretching band is evident between 3000 and 3500 cm⁻^1^, indicating the presence of hydroxyl groups. Aliphatic C-H stretching peaks are observed in the 2800–2950 cm⁻^1^ range, alongside a low-intensity carbonyl (C=O) stretching band between 1700 and 1800 cm⁻^1^. Peaks associated with unsaturation, specifically C=C stretching vibrations, appear as low-intensity signals between 1628 and 1700 cm⁻^1^. Additionally, the spectrum shows C-H bending vibrations between 1375 and 1450 cm⁻^1^ and C-O stretching vibrations within the 1000–1300 cm⁻^1^ range. Furthermore, out-of-plane C-H bending vibrations in alkenes are detected in the 650–1000 cm⁻^1^ region, providing a comprehensive insight into the molecular structure of the resin [29,30]. The FTIR spectrum of the KM-U resin, in addition to the characteristic acrylic peaks, shows two low-intensity bands at 3014 cm⁻^1^ (aromatic C-H stretching) and 1509 cm⁻^1^ (benzene ring stretching). These peaks likely originate from the presence of low amounts of benzoyl peroxide (BPO) usually used as a free-radical initiator in acrylic resin formulations [31].

Processing KM-U acrylic resin at an elevated temperature and pressure to produce cylindrical samples (R0) resulted in structural changes evident in the FTIR spectrum. The broadening of specific bands (e.g., 1375–1450 cm⁻^1^) and the splitting of others (e.g., 1234 cm⁻^1^) suggest increased crosslinking and hardening of the resin. The presence of hydroxyl (-OH) groups in the kaolin structure, together with functional groups identified in the structure of acrylic resin, could improve the interaction between these components. For instance, the hydroxyl groups on the kaolin surface can form hydrogen bonds with the functional groups of acrylic resins, enhancing interfacial adhesion between the two materials [32].

### 3.3. Thermal Analysis

Differential scanning calorimetry (DSC) measurements reveal exothermic or endothermic events, such as curing reactions or degradation processes, offering insights into the resin’s processing behavior and long-term durability [33].

**Figure 5:**
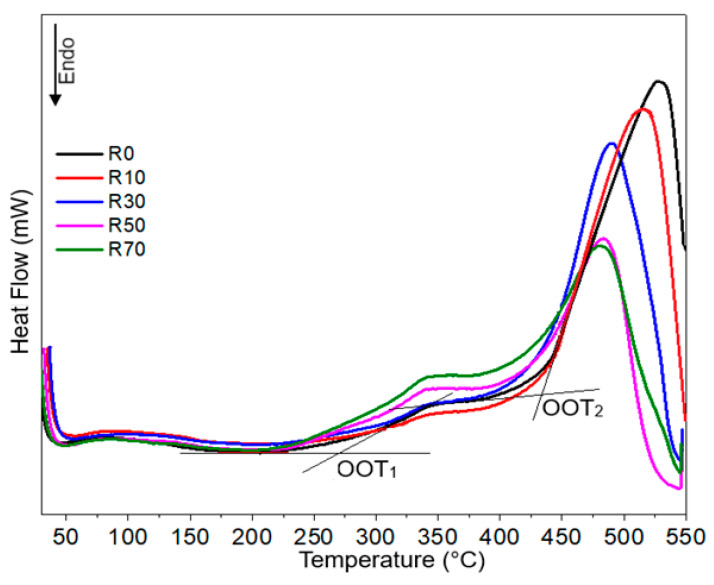
DSC curves recorded on KM-U resin with different filler amounts (air atmosphere, 10 °C/min).

DSC analysis under an oxidizing atmosphere provides critical information about a material’s thermo-oxidative stability [34]. The onset oxidation temperature (OOT), determined from the thermogram (Figure 5, Table 1), indicates the temperature at which the material starts to decompose due to oxidation. This parameter is essential for evaluating the material’s resistance to oxidation at elevated temperatures, and establishing the thermal limits of the material and its potential longevity under oxidative conditions (i.e., a higher OOT signifies greater stability against oxidative degradation) [35].

DSC analysis of KM-U/kaolin composites reveals two exothermic peaks due to oxidative decomposition of acrylic resins. The onset oxidation temperatures (OOT_1_ and OOT_2_) vary with kaolin content (Table 1). With up to 40% kaolin, both OOT values increase (from 266 °C to 277 °C for OOT_1_ and from 425 to 434 °C for OOT_2_), suggesting improved stability of the acrylic matrix. This effect may be due either to the neutralization of thermo-induced free radicals (i.e., thermo-oxidation is a free-radical chain reaction [36]) on the surface of kaolin particles (through hydroxyl groups) or due to a physical barrier to prevent oxygen access into the polymer matrix, slowing down the oxidation progress [37,38]. However, OOT values decrease as the kaolin concentration rises above 40% due to the kaolin particles’ greater agglomeration, which prevents them from effectively interacting with the acrylic resin matrix. In this way, oxidative degradation is accelerated by increased oxygen penetration into the material due to reduced interfacial bonding. At 70% kaolin concentration, OOT values are even lower than those of unmodified acrylic resin, highlighting that excessive kaolin content can compromise the composite’s thermal stability and oxidation resistance.

**Figure 6:**
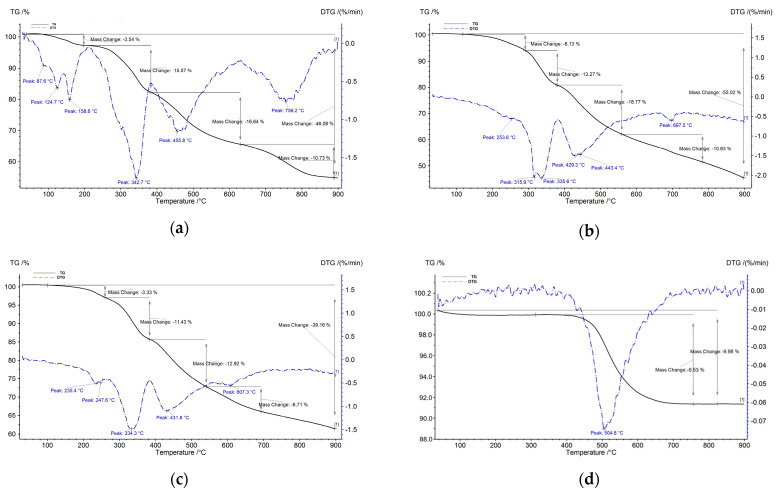
TG/DTG curves recorded on (**a**) KM-U resin powder; (**b**) R0; (**c**) R30; and (**d**) kaolin powder (N_2_ atmosphere, 10 °C/min).

TG curves (Figure 6) show a multi-stage decomposition process, which can be divided in three domains of temperature: below 205 °C; 205–650 °C; and over 650 °C. The first domain of temperature is observed only for KM-U resin powder and is characterized by two distinct peaks, accompanied by a cumulated mass loss of ~3.5%. The first peak observed between 60 and 120 °C is attributed to the evaporation of moisture present in the powder, and the second peak occurring between 140 and 205 °C is likely associated with the decomposition of BPO [39]. Interestingly, both of these peaks are absent in the DTG curves of the disk-shaped samples (R0–R70). The second temperature domain includes three main temperature peaks at ~250 °C, 335 °C, and 450 °C, with a total mass loss of 31–32% for KM-U resin powder and R0, and 24% for R30.

Generally, total mass loss decreases with an increase in kaolin content from 55.0% for R0 to 42.6% for R10, 39.16% for R30, and up to 34.3% for R70. Furthermore, a shift toward higher decomposition temperatures was observed with increasing kaolin content, likely due to a combination of physical and chemical factors that reduce heat transfer and delay the decomposition process within the composites, increasing their thermal stability [40]. In the case of kaolin powder (Figure 6d), the DTG peak observed between 450 and 600 °C corresponds to a dehydroxylation process (i.e., loss of OH groups from the structure associated with a loss mass of about 9.53%) and structural transformation of kaolin into metakaolin [40,41].

### 3.4. Electrical Properties

**Figure 7:**
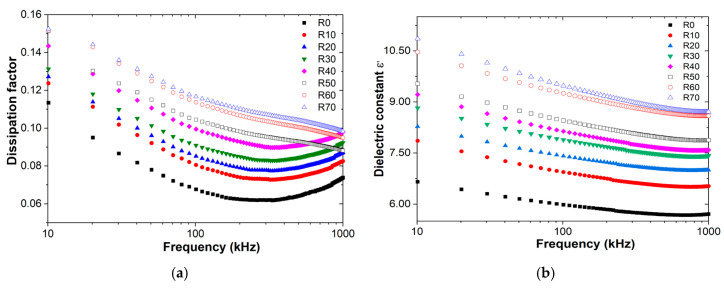
Dielectric spectroscopy measurements performed at room temperature on KM-U/kaolin composites: (**a**) dissipation factor and (**b**) dielectric constant.

Dielectric spectroscopy is a powerful technique used to characterize the dielectric properties of materials, such as the dissipation factor (tan δ) and dielectric constant, over a wide range of frequencies. The dissipation factor quantifies a material’s ability to convert electrical energy into heat, while the dielectric constant (or electrical permittivity) refers to a material’s ability to store electrical energy, both in an electric field [42,43]. When applied to KM-U/kaolin composites, dielectric spectroscopy reveals a clear relationship between dielectric properties and the amount of kaolin present. Figure 7 illustrates this dependence, showcasing how the dissipation factor and dielectric constant vary with frequency for different kaolin concentrations.

The addition of kaolin to acrylic resin generally increases the dissipation factor, with a direct correlation to kaolin concentration. This is likely due to kaolin’s higher dielectric loss compared to acrylic resin [44]. Analyzing the dissipation factor across frequencies, a significant decrease is observed up to 150 kHz, followed by a plateau region extending to 450 kHz. Beyond this point, a slight increase in dissipation factor is typically seen up to 1000 kHz. This behavior holds true for kaolin concentrations up to 40%. At higher concentrations, however, the dissipation factor exhibits a slightly different trend: a sharp decrease to 150 kHz, followed by a gradual decrease to 1000 kHz. These variations in dielectric properties between samples could be attributed to differences in interfacial polarization, which occurs at the boundaries between kaolin particles and KM-U resin [43].

It can also be observed that the dielectric constant exhibits a decreasing trend with increasing frequency, likely due to relaxation phenomena [43]. This, in turn, contributes to an increase in the dissipation factor, especially in composites containing up to 40% kaolin.

**Figure 8:**
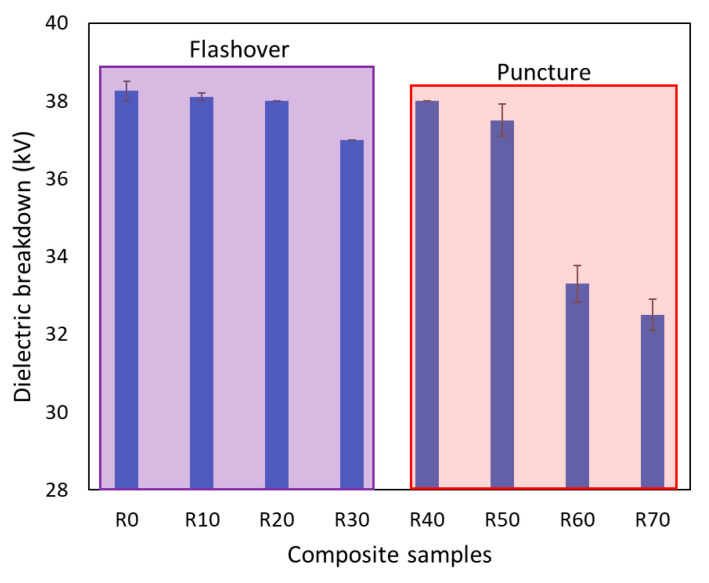
Dielectric breakdown strength of KM-U/kaolin composites (the error bars represent the standard deviation of three independent measurements).

Analyzing the breakdown strength results obtained on KM-U/kaolin composites (Figure 8), two types of breakdowns can be observed: flashover (i.e., the electric field between the electrodes causes a surface discharge along the surface of the composite) and puncture (i.e., the electric field causes a breakdown within the bulk of the composite material) [22].

The dielectric breakdown strength of acrylic resin composites containing kaolin exhibits a direct correlation with kaolin concentration. As kaolin content increases up to 30%, the primary failure mode is flashover. However, at higher kaolin concentrations, the breakdown mechanism shifts to material penetration.

The obtained results suggest that the KM-U/kaolin composites could be considered for applications where moderate dielectric properties are required: insulators, sensors, capacitors, etc.

### 3.5. Compression Strength

The resistance to compression of polymeric composites is influenced by various factors, including size and dispersion degree of the filler, interface quality, and matrix properties [45,46].

**Figure 9:**
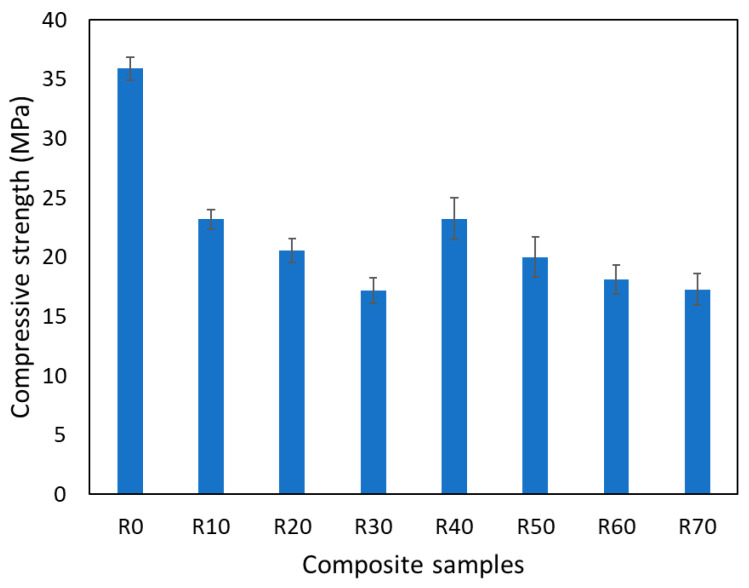
Tensile strength of KM-U/kaolin composites.

As observed in Figure 9, the tensile strength of the KM-U/kaolin composite samples generally decreases as the concentration of kaolin increases from 35.9 ± 0.95 MPa for the specimens without kaolin to 17.29 ± 1.30 MPa for the specimens with 70% kaolin. It can also be observed that the standard deviation increases with the increase in kaolin concentration. Even SEM analysis showed a good dispersion of kaolin powder in the KM-U structure; the interface between KM-U and kaolin particles is dependent on the amount of kaolin particles, influencing the effective load transfer between the components [6].

## 4. Conclusions

This study successfully developed acrylic resin/kaolin composites with kaolin concentrations ranging up to 70%, demonstrating the versatility of the acrylic resin matrix in accommodating a high filler content.

Characterization using various analytical techniques confirmed excellent kaolin dispersion throughout the resin, validating compatibility between the kaolin particles and the polymer matrix. Notably, the composites exhibited enhanced thermo-oxidative stability up to a kaolin concentration of 30%, without compromising their dielectric and mechanical properties. These findings suggest that the addition of kaolin, particularly at moderate levels, improves the thermal resilience of the material while maintaining its functional integrity, offering significant potential for applications in environments requiring thermal stability and dielectric performance.

However, further research is needed to optimize the composition and processing of these composites for specific applications. A preliminary result shows that the metakaolin structure form induces significative improvements in the dielectric and mechanical properties of acrylic resin. Factors such as particle size, distribution, and surface treatment will be investigated to tailor these properties. Additionally, studying the long-term stability and reliability of these composites under various environmental conditions is essential.

## Figures and Tables

**Table 1:** Decomposition temperatures of KM-U/kaolin composites (from DSC measurements in air).

Sample	OOT_1_ (°C)	OOT_2_ (°C)
R0	266.2	425.5
R10	272.0	430.3
R20	275.4	430.9
R30	276.4	432.2
R40	277.5	434.1
R50	272.5	425.1
R60	268.9	423.9
R70	250.8	422.4

## Data Availability

The data presented in this study are available on request from the corresponding author.
